# Immuno-Metabolic Modulation of Liver Oncogenesis by the Tryptophan Metabolism

**DOI:** 10.3390/cells10123469

**Published:** 2021-12-09

**Authors:** Véronique Trézéguet, Hala Fatrouni, Aksam J. Merched

**Affiliations:** MiRCade Team, BMGIC, INSERM U1035, University of Bordeaux, F-33000 Bordeaux, France; veronique.trezeguet-busquet@u-bordeaux.fr (V.T.); hala.fatrouni@u-bordeaux.fr (H.F.)

**Keywords:** tryptophan, kynurenin, metabolism, TDO2, IDO1, liver cancer, hepatoblastoma, immuno-oncology

## Abstract

Metabolic rewiring in tumor cells is a major hallmark of oncogenesis. Some of the oncometabolites drive suppressive and tolerogenic signals from the immune system, which becomes complicit to the advent and the survival of neoplasia. Tryptophan (TRP) catabolism through the kynurenine (KYN) pathway was reported to play immunosuppressive actions across many types of cancer. Extensive debate of whether the culprit of immunosuppression was the depletion of TRP or rather KYN accumulation in the tumor microenvironment has been ongoing for years. Results from clinical trials assessing the benefit of inhibiting key limiting enzymes of this pathway such as indoleamine 2,3-dioxygenase (IDO1) or tryptophan 2,3-dioxygenase (TDO2) failed to meet the expectations. Bearing in mind the complexity of the tumoral terrain and the existence of different cancers with *IDO1/TDO2* expressing and non-expressing tumoral cells, here we present a comprehensive analysis of the TRP global metabolic hub and the driving potential of the process of oncogenesis with the main focus on liver cancers.

## 1. Introduction to Liver Cancers

### 1.1. The Liver, an Extraordinary Organ with Multiple Functions

The liver is an essential organ involved not only in digestion but also in many other functions, among them immunotolerance [1]. Indeed, it is continuously exposed to antigens from food intake, gut microbiome, and possibly from pathogens. Kupffer cells, dendritic cells, and lymphocytes T participate in this tolerance.

Any dysfunction of the liver can therefore lead to important metabolic or immunological disorders, eventually threatening the survival of the organism and/or leading to liver tumorigenesis. In 2020, liver cancer ranked 6th in the world in terms of incidence (around 906,000 new cases) and 3rd in terms of mortality (around 830,000 deaths), with disparities between men and women as incidence and mortality rates were 2–3 times higher in men [2]. Liver cancer is expected to increase by 50% in the next 20 years with major geographical disparities in prevalence worldwide [3].

### 1.2. Hepatocellular Carcinoma in Adults

Hepatocellular carcinoma (HCC), also called hepatocarcinoma, is an adult primary liver cancer that develops from hepatocytes in 75–85% of cases [4] and, most of the time, in a diseased (chronic liver inflammation, fibrosis) and/or cirrhotic organ [5]. Other rarer forms of primary liver cancer can develop from cells in the bile ducts (cholangiocarcinoma, 10–15% of cases [4]) or, much more rarely, from blood vessels (epithelioid hemangioendothelioma). All causes of chronic liver disease are therefore directly or indirectly responsible for HCC. Among them the most frequent are the hepatitis B and C viruses, representing 56% and 20% of liver cancer deaths worldwide in 2012, respectively [6], alcohol abuse, aflatoxin B exposure, smoking, and non-alcoholic steatohepatitis (NASH). Diabetes and obesity are also risk factors of HCC. Hepatocarcinoma’s incidence has been steadily increasing for several years, due to the increase in viral infections or environmental toxins [7,8]. The etiology of HCC also lies in alterations of *TERT* (telomere reverse transcriptase), oncogenes and tumor suppressor genes (*P53*, etc.), and genes that lead to aberrant cell signaling pathways (Wnt-β-catenin pathway, etc.), in parallel with the development of cirrhosis and liver fibrosis [9].

In most cases, symptoms are absent in the early stages of the disease. By the time symptoms such as jaundice and weight loss occur, it is usually too late and the available treatment options extend survival by only a few weeks or months. This is why regular surveillance is recommended for patients with cirrhosis or chronic Hepatitis B or C Virus infections. Usually, it consists in an ultrasound scan, which may be combined with an evaluation of serum markers such as Apha-fetoprotein (AFP) or Glypican 3 (GPC3).

Staging is a requisite for proposing the best treatment strategy, and it must consider the underlying liver condition that will affect the evolution of the disease and dictate the applicability and effectiveness of the treatment. Around 18 staging classifications have been proposed, and are reviewed in [10]. The number and size of nodules and the presence of an underlying liver disease are the most common criteria but the presence of tumor and vascular invasions or the extent of liver involvement is also examined. Some of these staging systems are also of prognostic value. However, it appears that there is no real consensus when it comes to deciding which staging system offers the best classification. Though the main drivers of HCC are numerous, the molecular characterization was thus far of no help for stratification.

Surgical resection and liver transplantation are the best curative options, as they improve the survival chances, at least at an early stage of HCC. Only a quarter of the liver mass is necessary for a human organism to live normally. After resection, the liver has extraordinary regenerative capacities as new cells are rapidly generated to allow the remaining liver to grow back to its original size. Transcatheter arterial chemoembolization (TACE) or radioembolization (TARE) leading to tumor necrosis are proposed for intermediate stage patients. For the most advanced cases, multikinase inhibitors sorafenib or regorafenib can extend life by a few months [11,12]. Recently, FDA approved as second line-therapy for advanced HCC immune check-point inhibitors, alone or in combination [13,14], and anti-VEGF antibodies as reviewed in [14]. Attempts have been made to vaccinate with HCC specific antigens such as AFP, GPC3, or the multidrug resistance-associated protein MRP3. They were not associated with an increased overall survival [1]. The complexity of the role of the liver in self-defense and immunotolerance makes it difficult to envision immunotherapy treatment and underlines the urgent need for better understanding the immune environment in HCC to provide efficient treatments for longer survival and better quality of life.

Any treatment that may improve liver conditions, such as steatosis, fibrosis, NASH, could help reduce HCC risk. In this context, statins have been evaluated, as reviewed in [13,15]. Statins inhibit the rate-limiting step of cholesterol synthesis catalyzed by the 3-hydroxy-3-methylglutaryl CoA reductase (HMG-CoA reductase). Since their discovery in 1973, they are widely used as lipid lowering agents in the prevention of heart attack and stroke. They are now considered potentially chemopreventive thanks to several observational and experimental studies, although additional investigations are needed to meet these expectations. [13,15].

### 1.3. Hepatoblastoma in Children

Hepatoblastoma (HB) is a rare primary liver tumor with a worldwide incidence of 1.5 cases/million children per year. HB usually affects children under 5 years of age [16] for whom it is the most common liver tumor with the majority of cases appearing before 18 months. HB derives from parenchymal liver cells or hepatoblasts. It can occur sporadically, in children prematurely born or with a low birth weight, or in children with a family history of genetic diseases such as adenomatous polyposis or Beckwith-Wiedemann syndrome. Children who had hepatitis B at an early age or biliary atresia are at greater risk of developing the disease. HB can metastasize, mostly in the lung and the abdomen.

The mainstay of curative therapy based on clinical parameters and risk assessment [17] is a complete surgical resection. When not possible at first line, cisplatin and doxorubicin-based chemotherapies can help reduce the size of the tumor before resection. Four international groups have established standards of risk and treatment strategies that have helped to achieve a five-year survival rate of nearly 80%, up from 27% in the early 1990s [18], but the prognosis remains poor in 20% of cases. Hence, the side effects of chemotherapy and the management of the refractory cases remain to be improved.

Cisplatin is known to induce cytotoxicity, neurotoxicity, and nephrotoxicity, and cardiotoxicity of doxorubicin limits its use to treat HB. Other platinum derivatives such as carboplatin and oxaliplatin induced less cytotoxicity but were also less effective than cisplatin in HB treatment. These drugs induce irreversible DNA damage leading to cell death. The mechanisms of HB resistance to platinum derivatives could arise from DNA-unrelated effects of platinum and still need to be clarified [19]. Improvements in the understanding of HB are therefore of utmost importance. This implies answering several fundamental questions, including that of the HB driver genes and the evaluation of their drug-generability.

HB is associated with a significant increase in serum alpha-fetoprotein, the level of which is used as a prognostic and diagnostic marker [20]. β-catenin and the Wnt pathway were identified as key drivers of sporadic HB pathogenesis [21]. The Wnt/β-catenin has a key role in the development, regeneration, and zonation of the liver [22]. However, the list is far from close as cancers emerge as complex pathologies with different outcomes even though deriving from the same organ. For instance, in 2008, a 16-gene signature was proposed to discriminate two distinct HB subgroups [23], C1 and C2, and was further adopted.

More recently, a transcriptomic study was performed on tumor and non-tumor tissues from 22 hepatoblastoma patients. A 4-gene molecular signature was thus proposed to classify HB in three different types, C1, C2A, and C2B [24] based on the previous classification and not overlapping with the 16-gene signature. The C1 patients are at low risk. In the C2 types, which are of poorer prognosis, the C2A group is associated with high proliferation while the C2B type exhibits epithelial-mesenchymal transition features. Though different classifications are now internationally recognized, they are questioned because of the low number of patient samples and seem difficult to be validated in larger cohorts of patients. This stresses more than ever the need for a deeper understanding of the molecular biology of HB to propose other therapeutic options with less toxicity, notably for the chemoresistant HB. Research interests should focus on the crosstalk between two emerging hallmarks of cancer, metabolism and inflammation [25], whose better understanding may unravel new leads for such therapeutic perspectives.

## 2. Importance of Metabolism as a Feature for Cancer

Nutritional status is known to regulate cell signaling since the discovery in the 1970s that glucose levels were modifying hepatic ATP levels and leading to various consequences on organite structures and protein modifications such as phosphorylation [26]. For example, glutamine is one of the most consumed amino acids by mammalian cells and its shortage is compensated for by activating EGFR/Pak signaling in pancreatic cancer cells [27].

Metabolites, issued from nutrient consumption, are increasingly considered not only as end products or substrates of enzymatic reactions but also as signaling molecules that eventually govern and modulate many cellular processes among which protein function, cell signaling, gene expression, and intercellular communication [28]. Hence, lipids are not only components of cell membranes or used as energy storage, but have been shown to regulate signaling pathways such as those of PPARβ/∂, EGFR-KRAS, or NF-ĸB [29]. AMPK and mTORC1 signaling were also described as “master regulators” of cellular metabolism [29] and sensing of the status of both glucose and energy by AMPK and mediation of amino acid sensing by mTOR signaling are well documented nowadays [28].

Nutrient/metabolite sensing and signaling in cancer cells is an emerging field since the discovery of the oncometabolite 2-hydroxyglutarate (2HG). Mutations of the isocitrate dehydrogenases 1 and 2 (IDH1/2) were discovered as cancer drivers and it was observed that they occur in the catalytic site of IDH1/2, conferring to the mutant enzymes the ability to synthesize 2HG, responsible for triggering malignant transformation and leading to the development of some cancers [30]. More specific but global metabolic alterations in liver cancers are related to the modulation of the Warburg shift (from mitochondrial oxidative phosphorylation toward glycolysis and lactate production) and the upregulation of lipid catabolism. In depth analysis of all the changes in these catabolic pathways of lipid metabolism, e.g., lipogenesis and fatty acid oxidation, and the role of acetyl-CoA at the center of many metabolic pathways are reviewed elsewhere [15]. Metabolites from other nutrients such as L-tryptophan (TRP) are getting more attention for their contribution in oncogenic and immunomodulatory mechanisms. Globally, the induction of TRP metabolism in the majority of cancers is associated with pro-tumoral immunosuppressive microenvironment as well as tumor invasion and proliferation. In vitro and in vivo experiments and clinical trials have been conducted using inhibitors of this pathway. The results from all of these studies were not as conclusive as expected and encountered some setbacks. More specific details and discussions are presented in the following sections.

### 2.1. Tryptophan Metabolism: The KYN and 5-HT Pathways

**Figure 1:**
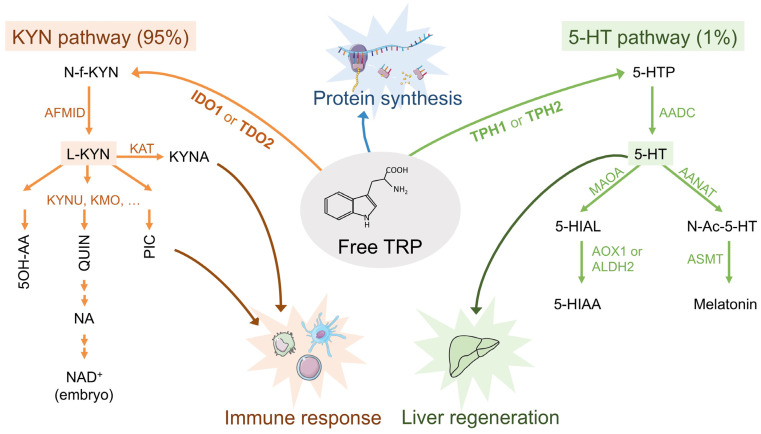
Free tryptophan cellular fate. The first and limiting step of the KYN pathway is catalyzed by tryptophan dioxygenase (TDO2) in the liver or indoleamine dioxygenase (IDO1/2) in immune cells. The first and limiting step of the 5-HT pathway is catalyzed by the tryptophan hydroxylase (TPH1). KYN pathway enzymes: IDO1/2, Indoleamine 2,3-dioxygenase 1/2; TDO2, tryptophan 2,3-dioxygenase; AFMID, arylformamidase; KYNU, kynureninase; KMO, kynurenine 3-monooxygenase; KAT, Kynurenine aminotransferase. KYN pathway metabolites: KYN, kynurenine; N-f-KYN, N-formylkynurenine; KYNA, Kynurenic acid; 5-OH-AA, 5OH-anthralinic acid; QUIN, quinolinic acid; PIC, picolinic acid; NA, nicotinic acid. 5-HT pathway enzymes: TPH1/2, Tryptophan 5-hydroxylase 1/2; AADC, Aromatic-l-amino-acid decarboxylase; MAOA, monoamine oxidase A; AANAT, aralkylamine N-acetyltransferase; AOX1, aldehyde oxidase 1; ALDH2, aldehyde dehydrogenase 2; ASMT, acetylserotonin O-methyltransferase. 5-HT pathway metabolites: 5-HTP 5-hydroxytryptophan; 5-HT, serotonin; 5-HIAL, 5-hydroxyindole acetaldehyde; 5-HIAA, 5-hydroxyindole acetate; N-Ac-5-HT, N-acetyl serotonin.

In mammals, TRP is a rare and essential amino acid provided only by dietary intake. In addition to being a component of proteins, TRP is metabolized through the kynurenine (KYN) or the serotonin (5-HT) pathway to lead to the production of physiologically active metabolites, such as kynurenic acid (KYNA) and NAD^+^ or serotonin (5-HT) and melatonin [31] (Figure 1). The first and limiting step of the synthesis of 5-HT is catalyzed by the tryptophan hydroxylase (TPH1). The first and limiting step of the KYN pathway is catalyzed by the tryptophan dioxygenase (TDO2) in the liver or the indoleamine dioxygenase (IDO1/2) in immune cells.

Under physiological conditions, the two pathways are unequal in their ability to degrade TRP as approximately 95% of free TRP is metabolized via the KYN pathway (of which 90% occurs in the liver) and about 1% through the 5-HT pathway. The KYN pathway itself comprises two main branches starting from KYN. The first one ends with KYNA (Figure 1). The second branch is favored under physiological conditions and leads to the production of 5-OH-anthralinic acid (5-OH-AA), the quinolinic and picolinic acids (QUIN and PIC), and of NAD^+^, a principal cofactor in cellular reactions related to energy metabolism, which is inspiring therapies in many diseases [32] (Figure 1).

IDO1 has been shown to be implicated in maternal tolerance towards ‘allogeneic concepti’ [33], in controlling autoimmune diseases [34,35] and chronic infection [36], as well as promoting tumor immune escape [37,38,39]. IDO1 was widely reported to be involved in immunosuppression of T lymphocytes. These actions were attributed to IDO1-caused depletions of TRP as well as to specific effects of TRP metabolites.

NAD^+^, the end-product of the KYN pathway, has a protective role in cancer, as shown in mice. Impaired TRP metabolism resulting in inhibition of de novo NAD^+^ synthesis in the liver promoted hepatic tumorigenesis through DNA damage [40]. In human gliomas, NAD^+^ produced de novo from TRP confers resistance to the oxidative stress induced by radio-chemotherapy, however, glioma cells and microglia cooperate to produce NAD^+^ [41]. Furthermore, in human cancer cells, IDO1 has been implicated in improving DNA repair and mediating resistance to treatments, such as the PARP inhibitor olaparib, γ-radiation, and the chemotherapeutic agent cisplatin, by production of NAD^+^ [42].

5-HT is not only an hormone and a neurotransmitter, but it also plays a key role in the digestion process and acts as a mitogen for different cell types, including hepatocytes, notably during liver regeneration following injury [43]. Melatonin, the end-product of the 5-HT pathway, controls circadian rhythmicity and could be the link between KYN and 5-HT pathways. Indeed it was shown that melatonin treatment of PC12 cells decreased the expression of AANAT, the first 5-HT transforming enzyme through melatonin production, while it induced the expression of IDO1 by upregulating the expression of the forkhead box protein O1 (FOX1), which binds to the IDO1 promoter and regulates its expression [44].

Recently, the expression of *TDO2*, the liver specific isoform, was studied at the protein level in HCC samples and other tumors thanks to new monoclonal antibodies [45]. TDO2 protein was found highly present in all of the HCC cells, confirming previous observations at the mRNA level [46] and thus the implication of TDO2 in liver tumorigenesis [47]. This was specific of HCC cells since TDO2 protein was barely detected in other tumor cells, but only in vascular structures in the tumors.

### 2.2. Tryptophan Metabolism: Relation to Inflammation

Immune cells are important sources and processing sites of KYN metabolites, as they express high levels of several enzymes of the KYN pathway such as IDO, KATs, and GPR35. KYNA is generally considered to be neuroprotective but has also been shown to be an agonist of the aryl hydrocarbon receptor (AhR) [48] and of an orphan G-protein-coupled receptor (GPR35) [49], thus regulating the immune and inflammatory responses. As the transcription factor AhR together with IDO1 and TDO2 are present in tumor cells, it has been proposed that KYN could have a dual role in promoting cancer invasion and immune escape [44,46].

Immunoregulatory dendritic cells (DCs) regulate T cell responses through IDO1 expression. It has been shown in 2005 that extreme TRP shortage leads to dysfunction of T-cells and antigen-presenting cells (APCs) through accumulation of uncharged tRNA^TRP^, which activates the GCN2 (general control non-depressible 2/eIF2α kinase 4), a stress-response kinase. GCN2-knockout T cell proliferation does not respond to inhibition by IDO-expressing DC from tumor-draining lymph nodes [50].

IFNγ strongly increases IDO1 expression in inflammatory sites to prevent immuno-pathologies or reactions. Activation of IDO1 in the KYN pathway has been reported to suppress the T cell immune response by activating or differentiating regulatory T lymphocytes (Treg), which can inhibit the proliferation of other T cells and thus prevent anti-tumor responses. The action of IDO1/TDO2 leads to the depletion of intracellular TRP and formation of immunosuppressive metabolites such as KYN, which accumulates in the extracellular space, and kynurenic, xanthurenic and cinnabarinic acids, which are all ligands of the aryl hydrocarbon receptor (AHR). Activation of AHR modulates the immune response by modifying the tolerance of T cells and by regulating dendritic cells [41]. The immunological effects of IDO1 are also manifested in macrophages, which can be classified into two types: M1 which are active and have inflammatory properties contributing to the elimination of liver cancer cells; M2 which are “alternatively” active and have anti-inflammatory properties promoting the propagation, proliferation, and invasion of cancer cells. IDO1 is highly expressed in M2 macrophages in the tumor microenvironment in 58% of hepatocellular carcinoma (HCC) cases [51]. The overactivity of IDO1 in these macrophages leads to a decrease in free TRP, which results in negative regulation of CD8+ T cells and inhibits Th17 differentiation. Metabolites of TRP, such as picolinic and 3-hydroxyanthranilic acids, inhibit T cell proliferation by unknown mechanisms [52].

At the genetic level, it has been shown that *IDO1* expression is under the control of Bin1. Bin1 loss elevates the STAT1- and NF-κB-dependent expression of *IDO1* [33], driving the escape of oncogenically transformed cells from T cell-dependent antitumor immunity [37].

Many of these data and information were effective in setting up the scientific mind around the TRP/KYN pathway as being the culprit of immunosuppression, which has to be silenced in order to trigger the immune killing machinery against cancer.

### 2.3. Tryptophan Metabolism: Expression Data in HB

**Figure 2:**
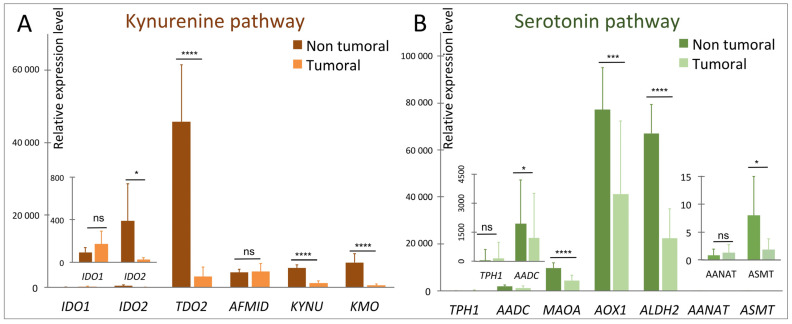
Variation of expression of some of the genes of the kynurenine (**A**) and serotonin (**B**) pathways in HB. See legend to Figure 1 for the abbreviations. *AANAT* and *ASMT*, coding both for melatonin-synthesizing enzymes, are barely detectable in both non-tumor and tumor samples while *TPH1* expression is increased in tumor samples. In HB, the 5-HT pathway would be reduced to 5-HTP production readdressing the question of the balance between KYN and 5-HT pathways in these tumors. (ns, not significant; *,1 × 10^−2^ < *p* < 5 × 10^−2^; ***, 1 × 10^−4^ < *p* < 1 × 10^−3^; **** *p* < 1 × 10^−4^).

**Figure 3:**
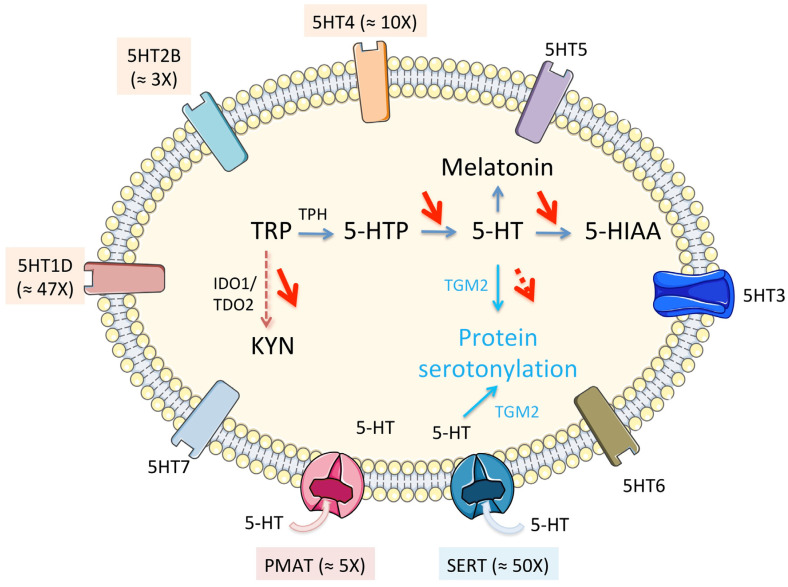
Schematic variations of the seven families of 5-HT receptors (5HT1 to 7), which are a group of G protein coupled receptors (all but 5-HT3) and ligand gated ion channel (5-HT3), and transporters (SERT and PMAT) in the cell membrane. SERT (*SLC64A*) is specific for serotonin while PMAT (*SLC29A4*) is a polyspecific organic cation transporter, notably for organic monoamines. PMAT is capable of transporting 5-HT through the plasma membrane, but at a much-reduced capacity as compared to that of SERT. In parentheses, ≈ xX indicates the extent of overexpression in HB compared to non-tumoral liver.

RNAseq analyses [24] show a perturbation of TRP metabolism in tumor tissue compared to non-tumor tissue manifested by (i) an increase in the expression of TRP transporters genes (notably *SLC1A5*), (ii) an overall reduction in the expression of enzymes of the KYN pathway (Figure 2A), notably in the *TDO2* gene of the limiting step enzyme, which is the liver-specific isoform, (iii) a non-significant increase in the expression of gene of the TRP to 5-HT conversion enzyme (*s*) but a significant decrease in the levels of the genes of the other enzymes of this pathway (Figure 2B) and, finally, (iv) a significant increase in three 5-HT receptor genes (*5HT1D*, *5HT2B*, and *5HT4*) (Figure 3).

The function of TDO2 in the normal liver is to downregulate systemic tryptophan. In HCC, its role would be the protection against immune-mediated tumor rejection. On the contrary, the metabolic context associated with HB could globally reflect an increased need for tryptophan by HB tumor cells, as we have observed that the catabolism of this amino acid in the KYN pathway seems largely reduced (Figure 2A). In addition, its conversion to 5-hydroxy-l-tryptophan (5-HTP) could be increased in parallel with a significant increase in some serotonin receptors and transporters at the tumor level (Figure 3).

SERT is the main 5-HT transporter in mammalian cells and the more specific but other transporters can carry 5-HT through the plasma membrane such as PMAT (Figure 3). The expression of SERT in the cell membrane is regulated by an intricate feedback mechanism involving 5-HT and, in some cases, the membrane-bound 5HT2B receptor. Circulating 5-HT acts to downregulate the expression of SERT on the plasma membrane, meaning that increased plasma concentration of 5-HT leads to a lower rate of uptake by cells [53].

5-HT signaling occurs through a family of membrane-bound receptors specific to 5-HT and named 5HT1 to 5HT7, with several subtypes (Figure 3). The 5HT2 family and the 5HT4 receptor are found primarily in peripheral tissues such as vascular smooth muscle and gastrointestinal tract (GIT) cells and their actions range from smooth muscle contraction in vasoconstriction and gastrointestinal motility to contraction of cardiac tissue causing tachycardia [54]. In healthy liver tissue, 5-HT receptor expression is low. There is evidence that the 5HT2B subtype is present in hepatocytes and is involved in hepatic cell proliferation. Its activation leads to a decrease in glucose uptake by the cells and an increase in lipolysis [55]. Its expression is increased around three times in HB [24]. The other overexpressed receptors are *5HT1D* (×47) and *5HT4* (×10) (Figure 3). Although relatively understudied in its relationship to cancer, the 5-HT1D receptor has been shown to activate the β-catenin pathway in colorectal cancer and influences metastasis [56]. In healthy tissue it is mainly found in the CNS and its action involves inhibiting neurotransmitter release, so the apparent upregulation in HB is interesting. As the β-catenin pathway is involved in 80% of HB it deserves to be further explored in the context of tryptophan metabolism.

To summarize, the unexpected change in the TRP metabolic pathway in the context of HB has the merit of making us reconsider in a more critical way the involvement of this pathway in oncogenesis and immunosuppression.

### 2.4. Tryptophan Metabolism: Other Less Known Downtream Metabolites

From a general viewpoint, the intermediates of amino acid metabolism have been shown to intersect with cellular signaling and this is reviewed in Wang et al. [57]. Activation of RAS/MAPK signaling has been frequently observed in various types of cancer, in line with its key role in promoting cell proliferation and survival. Interestingly, it is effectively suppressed by 5-hydroxyindoleaceticacid (5-HIAA), a product in tryptophan degradation, which could interact with membrane-bound receptors [58]. This metabolite is produced from 5-HT by MAOA and AOX1 in the cytosol or ALDH2 in the mitochondria (Figure 1). Interestingly, these enzymes are downregulated in HB, potentially preserving at least in part the RAS/MAPK signaling and thus tumor growth. 5-HIAA metabolism could potentially be targeted to totally suppress MAPK signaling by providing 5-HIAA precursor, for example.

The 5-HT pathway in HB could induce 5-hydroxytryptophan (HTP) over production. 5-HTP therapeutic effects include treatment of depression, chronic headache, and insomnia. This is another metabolite whose fate in HB could be investigated.

### 2.5. Tryptophan Metabolism: Also Involved in Protein Modification

**Figure 4:**
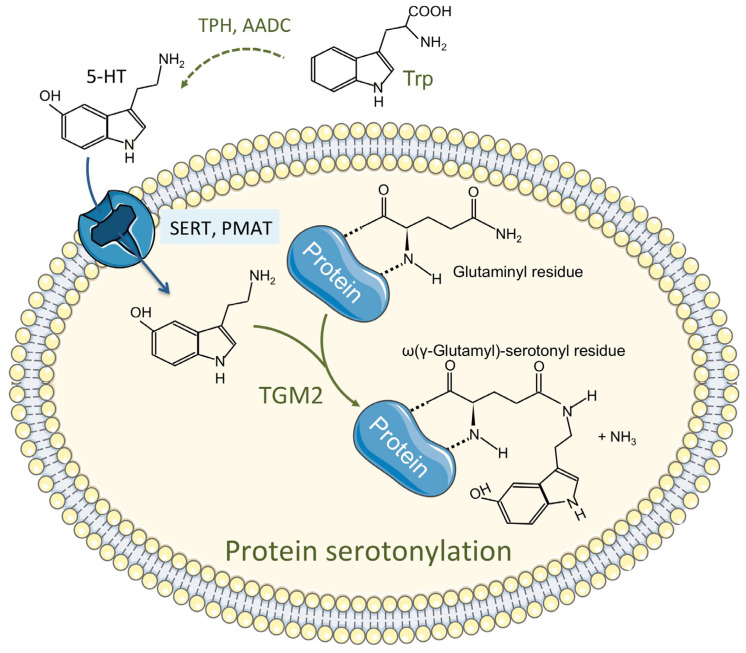
The serotonylation of proteins by TGM2.

A post-translational protein modification mechanism involving serotonin, called serotonylation, was discovered more than 60 years ago and has been since implicated in many diverse crucial intracellular processes. Serotonylation is a transamidation leading to the attachment of 5-HT to a protein glutaminyl residue catalyzed by transglutaminase 2 (TGM2) and the blood coagulation factor XIIIa (Figure 4). It results in a modification of the function of the recipient protein [59,60]. An updated list of serotonylated proteins includes fibronectin, fibrinogen, Rab-3a, Rab-4, Rab-27a, RhoA, Rac, Ras, histones, actins, myosins, Akt, and SERCA2 in various normal or cancerous cells [61]. However, so far nothing is known about the status of protein serotonylation in the liver.

*TGM2* is expressed all over the body, mainly within the cell, and its function depends on Ca^2+^ and goes well beyond serotonylation. A specific serotonylation motif has still not been defined. TGM2 is highly expressed in the non-tumor tissue of HB patients and its expression is decreased 3–7 times in the tumor tissues of the same patients [24]. Knowing that serotonylation modifies protein activity and recycling in the cell, TGM2 decrease in HB could have some implications in the regulation of the tumor cell metabolism. The target (s) of this post-translational modification needs to be identified to get a better understanding of the role of the intracellular levels of serotonin in tumor development.

### 2.6. Tryptophan Metabolism in Pediatric Liver Cancer: Not the Same as in Adult Liver Cancers

Considering the metabolo-pathological variations of HB relative to HCC, the idea of inhibiting the TRP/KYN pathway in order to improve immunotherapy responses does not seem relevant and even counterproductive in an HB-related pathological context. Not surprisingly, there have been failures in clinical trials with IDO inhibitors such as epacadostat. Some researchers and investigators are pushing harder and suggest the use of a combination of TDO2/IDO or COX-2/IDO1 inhibitors [62]. We will therefore see further clinical disappointment if the metabolic crossroads around TRP are not well clarified.

## 3. Therapeutic Targets within the TRP Pathways

### 3.1. Ongoing Clinical Trials

Given the close relationship between KYN metabolites and inflammatory responses, several drugs targeting COX-2, IDO1, IDO2, or TDO2 are already being tested in clinical trials for the treatment of some cancers ([63] and Table 1), some of which have already been completed. The results of the most advanced clinical trial (Phase 3) using an IDO1 activity inhibitor, epacadostat (trial ECHO-301) have been disappointing for the treatment of metastatic melanoma [62]. Indeed, the combination of epacadostat with pembrolizumab (a reference anti-PD1 treatment in immunotherapy) does not show any beneficial effect of this inhibitor and does not appear to act on the immunosuppressive microenvironment. This failure calls into question the involvement of IDO1 in immunosuppression as well as the usefulness of the IDO1 inhibition strategy. IDO1 is involved in TRP degradation but also in cell signaling and the latter should be targeted along with the catalytic activity [62]. Some researchers have suggested that the immuno-modulatory effect of the inhibitors in preclinical studies may be the consequence of an off-target and independent action of IDO1 inhibition [64]. However, using the IDO inhibitor 1-methyl-tryptophan (1-MT) in association with diverse chemotherapeutic agents, such as cisplatin, cyclophosphamide, and doxorubicin, could effectively promote the regression of breast tumors known to be refractory to chemotherapy [37].

### 3.2. Other IDO Inhibitors

Other drugs targeting the key enzyme of the kynurenine pathway IDO1 have been used in vitro and/or in vivo and are listed in Table 2. 1-MT exists as two stereoisomers, 1-D-MT and 1-l-MT and IDO1 is the preferential target of 1-l-MT, while 1-D-MT, which has been used in clinical trials, preferentially inhibits IDO2 [65,66]. Despite the bulk of evidence supporting a role for IDO1 in promoting tumor formation and tumor immune escape, there have been clinical studies showing an anti-tumor effect of IDO1 by the intermediate of IFN-γ, which was effective in the therapy of ovarian carcinoma and bladder cancer [67,68,69]. At the preclinical level, a study on pre-immunized mice with tumor antigen has shown that the tumor volume was reduced with 1-methyl-tryptophan (1-MT) and a regression of established breast cancer was observed when 1-MT was combined with chemotherapy [39]. However, other studies have shown that *IDO1* expression in tumors positively correlated with progression-free survival and long-term survival [69,70].

In term of mechanism, 1-D-MT inhibits p38 MAPK phosphorylation, preventing the increase of IDO1 mRNA and therefore the increase in KYN production. Additionally, it contributes to the inhibition of JNK signaling, which attenuates the expression of *IDO1* mRNA and as a consequence the release of KYN [71]. 1-l-MT, the stereoisomer of 1-D-MT was recently reported to suppress the IFN-γ-induced expression of *IDO1* in mouse rectal carcinoma cells [71]. The TRP-catabolizing enzyme IDO2 was not induced by 1-D-MT. This information sheds light on the different regulation of *IDO1* and *IDO2* at the tran scriptional level. Uncertainty remains about the clinical significance of *IDO1* expression in tumors. However, at some point, it is relevant to link the anti-tumor outcome of 1-D-MT to the induction of *IDO1* by IFN-γ.

Methyl-thiohydantoin-tryptophan (MTH-TRP), another IDO inhibitor, has been discovered and is 20-fold more potent than 1-MT. Its sidechain is a mimetic of the amino acid backbone of tryptophan and it is more soluble in water than 1-MT but it is also more rapidly cleared from serum, both compounds being orally bioavailable [37].

In conclusion, *IDO1*’s expression is known to be immunosuppressive and may be involved in tumor immune escape, but it has also been implicated in direct anti-tumor effects. More studies are needed to better understand the role of IDO1 and IDO2, the implication of their inhibition and to determine whether a correlation exists between these enzymes and other important enzymes of the TRP metabolism such as TDO2.

### 3.3. Other TDO2 Inhibitors

Several studies have reported functional TDO2 expression in various human cancers including bladder, melanoma, and hepatocellular carcinoma [74]. In a preclinical mouse model, it has been shown that *TDO2* expression by tumor cells prevented their rejection by immunized animals [75]. Interestingly, this type of immunosuppression prevents allograft rejection usually observed after liver transplantation [75]. TDO2 promotes tumor progression through the production of kynurenine, which is an endogenous ligand of the aryl hydrocarbon receptor (AHR), known to be involved in increased tumor cell survival and motility, and reduced anti-tumor immune responses. Altogether, these data suggest that pharmacological inhibition of TDO2 could reactivate the immune system and promote tumor destruction. So far, several compounds were reported as TDO2 inhibitors such as 680C91 [72], which has poor bioavailability and poor solubility, replaced by LM10, which has high bioavailability and solubility [73].

In another study, ~2800 compounds from the Library of National Cancer Institute USA were screened, among which seven were potent inhibitors of TDO2, with inhibition rates in the nanomolar or low micromolar ranges, and six of them inhibited both IDO1 and TDO2. All these inhibitors have antitumor characteristics on different cancer cell lines [74] (Table 2). NSC 26326 or β-lapachone, which is a topoisomerase I inhibitor, was the strongest IDO1/TDO2 inhibitor, with Ki = 97 ± 14 nM for TDO2 and 30–70 nM for IDO1 [74].

This pharmacological approach may be further extended to a wider range of eligible tumors before entering into clinical studies.

In conclusion, according to some preclinical data pharmacological inhibition of TDO2 and IDO1 might represent a safe and efficient approach to treat cancer by promoting tumoral destruction by the immune system, and consequently potentiating cancer immunotherapies whether it is a single shot inhibition or a two-shot inhibition (IDO1 and TDO2).

## 4. Conclusions

The expression of *IDO1* in the majority of cancers is related to immunosuppression, which promotes tumorigenesis, while the expression of *TDO2* is responsible for tumor invasion and proliferation. In hepatoblastoma, the role of these two key enzymes of the kynurenine pathway is unknown, as their expression is largely diminished, unlike in other cancers.

In vitro, in vivo, and clinical trials have been undertaken to develop inhibitors of these genes or of others related to the functioning of IDO1 and TDO2. Some of them have been successful, others were not, like the famous combination of epacadostat with an immune checkpoint inhibitor, anti-PD-1/PD-L.

Clinical trials have been completed for inhibitors alone or combined with chemotherapy or monoclonal antibodies. Further studies are needed to more comprehensively evaluate the TRP metabolic node, taking into account the role of the kynurenine pathway and specifically the role of key enzymes in this pathway, in pediatric liver cancer oncogenesis. Will the absence of these enzymes in hepatoblastoma be the cause of immunosuppression and/or the driver of other protumoral activities, unlike what happens in other types of cancer? Further in-depth investigations are required.

## Figures and Tables

**Table 1:** List of clinical trials with anti-cancer drugs targeting COX-2, IDO1, or TDO2 in various cancers. Taken from https://clinicaltrials.gov/, last accessed 31 August 2021.

Target	Inhibitor	Strategy (Combination)	NCT Number	Phase	Type of Cancer	Status
IDO1	1-methyl-D-tryptophan	alone	NCT00739609	1	breast cancer, lung cancer, melanoma, pancreatic cancer, solid tumors	terminated
IDO1	GDC-0919 (navoximod)	alone	NCT02048709	1	solid tumors	completed
FIXEDIDO1	LY3381916	LY3300054 (anti-PD-L1 checkpoint antibody)	NCT03343613	1	non-small cell lung cancer, renal cell carcinoma, triple negative breast cancer	terminated
IDO1	NLG802	alone	NCT03164603		advanced solid tumors	completed
IDO1	BMS-986205	nivolumab + ipilimumab	NCT03459222	2	advanced cancer	recruiting
IDO1	BMS-986205	alone	NCT03695250	1	liver cancer	active, not recruiting
		+ nivolumab		2		
IDO1	BMS-986205	nivolumab + temozolomide + radiotherapy	NCT04047706	1	glioblastoma	recruiting
IDO1	epacadostat (INCB024360)	itacitinib (JAK inhibitor) + INCB050465 PI3K-delta inhibitor	NCT02559492	1	solid tumors	terminated
IDO1	epacadostat (INCB024360)	nivolumab + anti-GITR monoclonal antibody MK-4166 + ipilimumab	NCT03707457	1	glioblastoma	terminated
IDO1	epacadostat (INCB024360)	ALVAC(2)-NY-ESO-1 (M)/TRICOM vaccine	NCT01982487	1	epithelial ovarian, fallopian tube, peritoneal cancer	withdrawn
		alone		2		
IDO1	epacadostat (INCB024360)	DEC-205/NY-ESO-1 fusion protein CDX-1401 + Poly ICLC	NCT02166905	2	fallopian tube carcinoma, ovarian carcinoma, primary peritoneal carcinoma	completed
IDO1	epacadostat (INCB024360)	pembrolizumab	NCT03414229	2	sarcoma	active, not recruiting
IDO1	epacadostat (INCB024360)	pembrolizumab	NCT03432676	2	advanced pancreatic cancer	withdrawn
IDO1	epacadostat (INCB024360)	cyclophosphamide	NCT02785250	2	ovarian cancer	active, not recruiting
IDO1	epacadostat (INCB024360)	ipilimumab	NCT01604889	2	metastatic melanoma	terminated
IDO1	epacadostat (INCB024360)	azacitidine (DNA methyltransferase inhibitor) + pembrolizumab	NCT02959437	2	metastatic cancer	terminated
		INCB057643 + pembrolizumab				
		INCB059872 + pembrolizumab				
IDO1	epacadostat (INCB024360)	pembrolizumab + cisplatin + cetuximab + carboplatin + 5-fluorouracil	NCT03358472	3	head and neck cancer	active, not recruiting
IDO1	epacadostat (INCB024360)	pembrolizumab + sunitinib + pazopanib	NCT0360894	3	renal cell carcinoma	active, not recruiting
IDO1 and TDO2	DN1406131	alone	NCT03641794	1	advanced solid tumors	recruiting
IDO1 and TDO2	HTI-1090	alone	NCT03208959	1	advanced solid tumors	completed
TDO2 and IDO1	DN1406131	alone	NCT03641794	1	advanced solid tumors	unknown
COX2	celecoxib 200 mg capsule	alone	NCT03896113	2	endometrial carcinoma	recruiting

**Table 2:** List of molecules or drugs tested as inhibitors of IDO1 and/or TDO2 in vitro, some of which are from the library of the National Institute of Cancer.

Target	Drug	Development Stage	Observations	Characteristics
IDO1, P38/MAPK pathway, JNK pathway	1-l-MT (1-methyl-l-tryptophan) [63,64,71]	in vitro, in vivo	delays tumor outgrowth when combined with chemotherapeutic agents	bioavailable
IDO1 inhibitor	MTH-TRP (methyl-thiohydantoin-trypt-ophan) [37]	in vitro, in vivo	delays tumor outgrowth when combined with chemotherapeutic agents	20-fold more potent than 1-MT, more rapidly cleared from serum, bioavailable
TDO2 inhibitor (mRNA level)	680C91 [72]	in vitro		poor bioavailability, poor solubility
TDO2 inhibitor	LM10 [73]	in vitro, in vivo		high bioavailability, high solubility
IDO1 and TDO2 inhibitor	NSC 26326 or β-lapachone [74]	in vitro	more potent inhibitor of TDO2 than IDO1	natural quinone isolated from lapacho tree; topoisomerase I inhibitor
IDO1/TDO2 inhibitor inhibits DNA synthesis JNK pathway inducing upregulation of death receptors	mitomycin C [74]	in vitro	8-fold more potent inhibitor of TDO2 than IDO1	active on 74 different tumor cell lines
TDO2 inhibitor	NSC 36398 (dihydroquercetin, taxifolin) [74]	in vitro	potent inhibitor of TDO2; no inhibition of IDO1	natural flavonoid with low toxicity
IDO1 and TDO2 inhibitor	NSC 267461 (nanaomycin A) [74]	in vitro	more potent inhibitor of TDO2 than IDO1	naphtoquinone based antibiotic; active on 59 cancer cell lines
IDO1 and TDO2 inhibitor	NSC 111041 [74]	in vitro	more potent inhibitor of TDO2 than IDO1	active on colon and breast cancer cell lines
IDO1 and TDO2 inhibitor	NSC 255109 [74]	in vitro	strong inhibitor of both IDO1 and TDO2	geldanamycin derivative; active on 65 different cell lines
IDO1 and TDO2 inhibitor	NSC 261726 (3-deazaguanine) [74]	in vitro	stronger inhibitor of TDO2 than IDO1	active on leukemia tumor cell lines

## Data Availability

Data from Figure 2 are from Hooks et al. (2018) [24]. Data from Table 1 were taken from https://clinicaltrials.gov/, (last accessed 31 August 2021).
